# Cystinuria Associated with Different *SLC7A9* Gene Variants in the Cat

**DOI:** 10.1371/journal.pone.0159247

**Published:** 2016-07-12

**Authors:** Keijiro Mizukami, Karthik Raj, Carl Osborne, Urs Giger

**Affiliations:** 1 Section of Medical Genetics, School of Veterinary Medicine, University of Pennsylvania, Philadelphia, PA, United States of America; 2 Veterinary Clinical Sciences, College of Veterinary Medicine, University of Minnesota, Saint Paul, MN, United States of America; Texas A&M University, UNITED STATES

## Abstract

Cystinuria is a classical inborn error of metabolism characterized by a selective proximal renal tubular defect affecting cystine, ornithine, lysine, and arginine (COLA) reabsorption, which can lead to uroliths and urinary obstruction. In humans, dogs and mice, cystinuria is caused by variants in one of two genes, *SLC3A1* and *SLC7A9*, which encode the rBAT and b^o,+^AT subunits of the b^o,+^ basic amino acid transporter system, respectively. In this study, exons and flanking regions of the *SLC3A1* and *SLC7A9* genes were sequenced from genomic DNA of cats (*Felis catus*) with COLAuria and cystine calculi. Relative to the Felis catus-6.2 reference genome sequence, DNA sequences from these affected cats revealed 3 unique homozygous *SLC7A9* missense variants: one in exon 5 (p.Asp236Asn) from a non-purpose-bred medium-haired cat, one in exon 7 (p.Val294Glu) in a Maine Coon and a Sphinx cat, and one in exon 10 (p.Thr392Met) from a non-purpose-bred long-haired cat. A genotyping assay subsequently identified another cystinuric domestic medium-haired cat that was homozygous for the variant originally identified in the purebred cats. These missense variants result in deleterious amino acid substitutions of highly conserved residues in the b^o,+^AT protein. A limited population survey supported that the variants found were likely causative. The remaining 2 sequenced domestic short-haired cats had a heterozygous variant at a splice donor site in intron 10 and a homozygous single nucleotide variant at a branchpoint in intron 11 of *SLC7A9*, respectively. This study identifies the first *SLC7A9* variants causing feline cystinuria and reveals that, as in humans and dogs, this disease is genetically heterogeneous in cats.

## Introduction

Cystinuria, originally described as one of the first inborn errors of metabolism in 1910, is characterized by a selective proximal renal tubular defect affecting cystine, ornithine, lysine, and arginine (COLA) reabsorption, which can lead to cystine crystals and urolithiasis [1]. Cystinuria occurs in humans (OMIM #220100), dogs (OMIA 000256–9615) [2–4], cats (OMIA 001878–9685) [5–7], primates [8], ferrets [9], mice [10], and some wild carnivores including wolves [8,11], foxes [8], caracals [12,13], and servals (Giger et al. unpublished, 2014).

In humans and dogs, cystinuria is caused by variants in either *SLC3A1* or *SLC7A9*, genes that encode the rBAT and b^o,+^AT subunits of the b^o,+^ basic amino acid transporter system, respectively [1–3]. The *SLC3A1* gene encodes rBAT, a luminal globular protein with a single transmembrane tail, whereas the *SLC7A9* gene encodes b^o,+^AT protein, a typical intramembrane transporter protein. The COLA amino acid transporter was originally thought to be a heterodimer, but is likely a heterotetramer formed from 2 heterodimers of rBAT and b^o,+^AT [14]. Cystinuria can be inherited in humans as an autosomal recessive (type IA and IB) and dominant (type IIA and IIB) trait due to *SLC3A1* (IA and IIA) and *SLC7A9* (IB and IIB) variants, respectively. In dogs, a similar heterogenity has been observed, albeit only one *SLC7A9* variant has been identified [2,3,15]. However, dogs have an additional androgen-dependent type (type III) cystinuria, which only manifests in mature intact males and is corrected by medical and surgical castration [3,16]. The molecular basis for type III is still unknown, although markers have been identified in some canine breeds (Henthorn et al. unpublished, 2016).

Feline cystinuria was first documented in a single case report in 1991 [5]. Subsequently, clinical features in 18 cystinuric non-purpose-bred domestic short-haired (DSH) and purebred cats (*Felis catus*) were summarized [7]. Cystinuria occurs less commonly in cats than in humans [1] and dogs based upon laboratory urolithiasis surveys. Cystine calculi represent only 0.1% of all feline uroliths in the United States and Canada compared with 0.3–1% of canine uroliths [17–19]. We have recently identified the first *SLC3A1* gene variant (p.Arg448Trp) in one severely cystinuric DSH cat in the United States [6]. We report here the first *SLC7A9* variants responsible for causing cystinuria in cats. These cats appear to have severe cystinuria with juvenile to mid-adult urinary tract signs and obstruction and exhibit likely neurologic metabolic derangements, presumably secondary to arginine deficiency, as arginine is an essential amino acid for cats [20].

## Results

### Signalments, urinary calculi and urinalysis

Based upon urinary crystal, calculi, nitroprusside (cystine) and/or amino acid analyses we found 7 cats with cystinuria, including 2 purebred (#1A, 1B), 2 non-purpose-bred domestic short- (DSH) (#4, 5), 2 medium- (DMH) (#1C, 3) and 1 long-haired (DLH) (#2) cats. We also included the previously reported DSH cat with an *SLC3A1* variant for comparison (Table 1) [6]. These cats were juvenile to middle-aged when clinical signs first appeared, and there was an equal distribution of females and males. All cats were either prepubertal or neutered before urinary cystine crystal and calculi formation occurred. Uroliths were composed of 100% cystine in all cats except one, in which prior catheterization likely promoted a secondary struvite (20%) admixture. One cat (#2) showed repeatedly severe cystine crystalluria that started at 4 months of age, but calculi did not form before its death, one month later. The urinary nitroprusside test result was strongly positive (3+/4+) for all cats, except for one (#4) that was only weakly positive (1+). The urinary cystine and COLA concentrations were high in 3 of 6 cats tested (#1A, 1B, 2), while others had concentrations in the normal range despite having had cystine crystal and calculi formation (Table 1).

**Table 1:** Comparison of clinical, clinicopathological, urinary metabolic, and genetic findings in cystinuric cats.

ID	Signalment					Urinary Test								Likely causative Variant^3^
	Breed	Sex	Location (USA/Canada)	Age (years)		NP test^2^	Cystine Crystal	Cystine Urolith	Urine (μmol/g Creatinine)					
				At 1st Urolith Submission	At Introduction to PennGen				Cystine	Ornithine	Lysine	Arginine	Sum of COLA	
1A	Maine Coon	FS	Pennsylvania	4.58	5	4+	+	100%	819	59	989	133	2000	p.Val294Glu
1B	Sphynx	MN	Florida	1.17	3	3+	+	80% (Struvite 20%)	303	256	3669	883	5111	p.Val294Glu
1C	DMH	MN	Ontario	NA	4	3+	+	100%	109	15	211	49	384	p.Val294Glu
2	DLH	F	New York	NA	0.33	4+	+	NA	364	273	1019	1353	3009	p.Thr392Met
3	DMH	FS	Ontario	NA	4.42	NA	+	100%	NA	NA	NA	NA	NA	p.Asp236Asn
4	DSH	FS	Washington	4.25	4.83	1+	-	100%	8	19	310	26	363	c.1233+1G>A
5	DSH	MN	California	0.33	2.5	4+	+	100%	37	7	72	18	134	c.1409-30A>G
6^1^	DSH	MN	Wyoming	0.33	0.42	NA	+	100%	NA	NA	NA	NA	NA	p.Arg448Trp^1^

Amino acids are depicted with symbols recommended by IUPAC-IUB. DSH, domestic short-haired cat; DLH, domestic long-haired cat; DMH, domestic middle-haired cat; M, male; MN, male castrated; F, female; FS, female spayed;

^1^cystinuric cat previously reported with *SLC3A1* variant [6]

^2^NP, nitroprusside test (score ranged from 0 to 4+)

^3^variants in *SLC7A9* (ID #1 to #5) and *SLC3A1* (ID #6) genes;

NA, not available.

### Variant analyses

**Fig 1:**
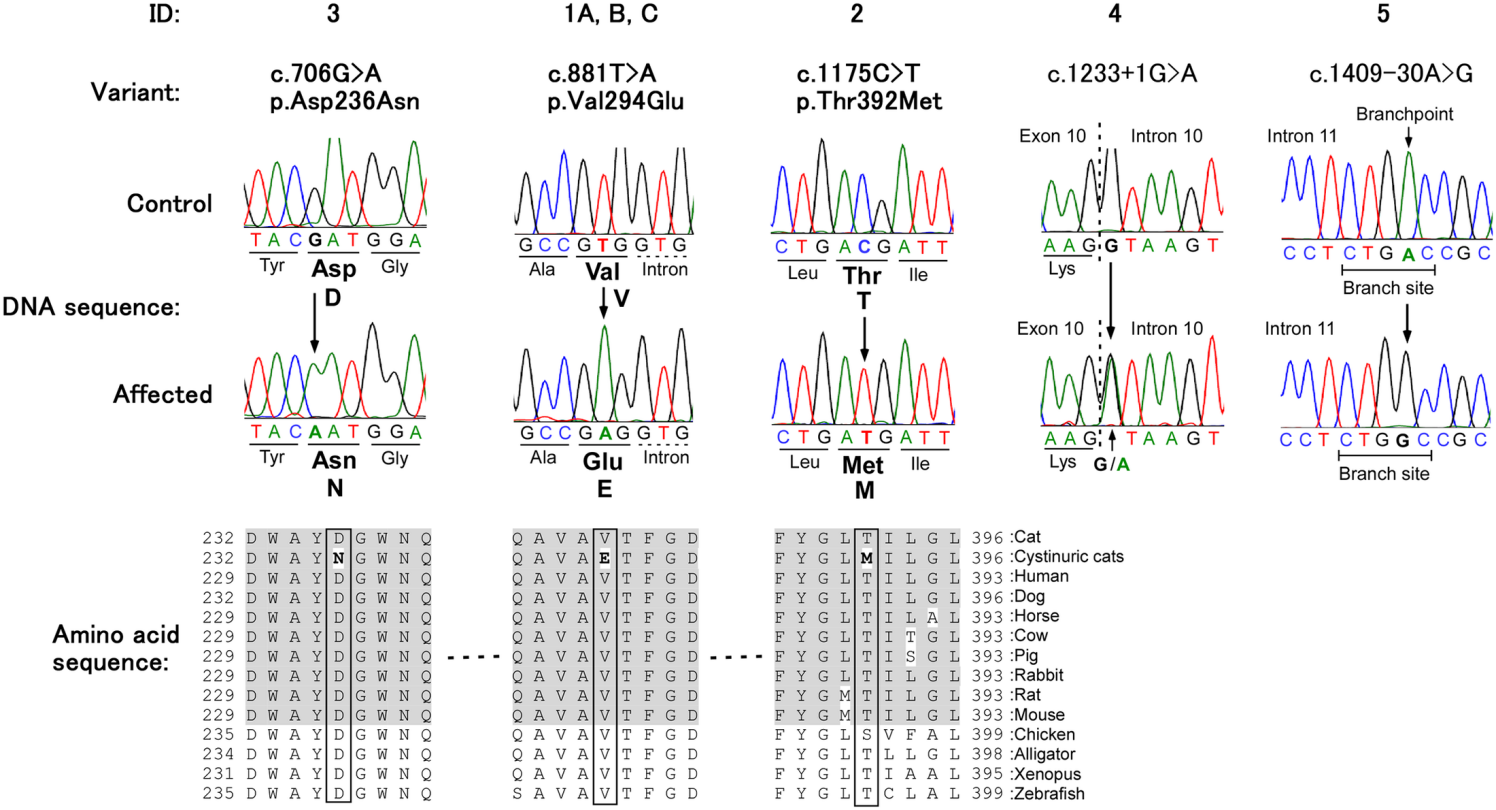
Genomic DNA sequencing chromatograms of regions of exons 5, 7 and 10 and splice site of intron 10 and putative branch site of intron 11 of the *SLC7A9* gene from a healthy and cystinuric cats, and the amino acid sequence homology of the feline *SLC7A9* gene among species and the site of missense variants (box). The homozygous single nucleotide substitutions in exons 5, 7 and 10 are unique to the cystinuric cats and are predicted to change the amino acids from aspartic acid (Asp, D) to asparagine (Asn, N), valine (Val, V) to glutamic acid (Glu, E) and threonine (Thr, T) to methionine (Met, M), respectively. Additionally, a heterozygous and homozygous single nucleotide substitution are identified at the donor splicing site of intron 10 and putative branchpoint of intron 11 in two different cystinuric cats. Variants identified in cystinuric cats are bolded and the conserved areas among mammals are shaded.

When compared to the feline reference genome sequence (GenBank accession no.: AANG03028500.1 and AANG03028501.1) [21] and the *SLC7A9* sequence of a normal healthy cat sequenced here, there were 10 exonic and 41 noncoding variants in the *SLC7A9* genes of the 7 cystinuric cats sequenced in this study (S2 Table). The cystinuric cat with the previously reported *SLC3A1* variant had, as expected, an entirely normal *SLC7A9* sequence. However, in addition to several other *SLC7A9* variants, the additional 6 cystinuric cats studied here bore 3 missense variants and 1 splicesite and 1 branchpoint variants (Fig 1). One cystinuric Maine Coon (#1A) and Sphinx (#1B) cat from different geographic areas were homozygous for the same *SLC7A9* point variant (c.881T>A) which causes a valine to glutamic acid substitution (p.Val294Glu) in the feline b^o,+^AT protein. One cystinuric DLH cat (#2) was homozygous for c.1175C>T variant causing a threonine to methionine substitution (p.Thr392Met) in b^o,+^AT. A cystinuric DMH cat (#3) was homozygous for c.706G>A in the *SLC7A9* gene which causes an aspartic acid to asparagine substitution (p.Asp236Asn) in b^o,+^AT. These 3 amino acid residues are highly conserved and reside in a conserved region in vertebrates as evolutionarily distant as the zebrafish (Fig 1). Furthermore, most modeling programs predict deleterious effects in the function of b^o,+^AT protein for all 3 observed amino acid changes in cystinuric cats as well as humans (Table 2).

**Table 2:** Function prediction for non-synonymous variants with multiple *in silico* tools.

Programs	*SLC3A1*						*SLC7A9*					
	c.1342C>T		c.1798C>G		c.1846A>G		c.706G>A		c.881T>A		c.1175C>T	
	Cat	Human	Cat	Human	Cat	Human	Cat	Human	Cat	Human	Cat	Human
	R448W	R452W^1^	P600A	L604A	I616V	I620V	D236N	D233N	V294E	V291E	T392M	T389M^1^
SIFT	***Deleterious***	***Deleterious***	Tolerated	Tolerated	Tolerated	Tolerated	***Deleterious***	***Deleterious***	***Deleterious***	***Deleterious***	***Deleterious***	***Deleterious***
PROVEAN	***Deleterious***	***Deleterious***	Neutral	Neutral	Neutral	Neutral	***Deleterious***	***Deleterious***	***Deleterious***	***Deleterious***	***Deleterious***	***Deleterious***
PolyPhen2-HVar	***Probably Damaging***	***Probably Damaging***	Benign	Benign	Benign	Benign	Benign	Benign	***Probably Damaging***	***Probably Damaging***	***Probably Damaging***	***Probably Damaging***
Pmut	Neutral	***Pathological***	Neutral	Neutral	Neutral	Neutral	Neutral	Neutral	***Pathological***	***Pathological***	***Pathological***	Neutral
SNAP	***Non-neutral***	***Non-neutral***	Neutral	Neutral	Neutral	Neutral	Neutral	Neutral	***Non-neutral***	***Non-neutral***	***Non-neutral***	***Non-neutral***
Align-GVGD^2^	***C65***	***C65***	*C25*	***C65***	*C25*	*C25*	C15	C15	***C65***	***C65***	***C65***	***C65***
PANTHER	***Deleterious***	***Deleterious***	***Deleterious***	***Deleterious***	Neutral	Neutral	***Deleterious***	***Deleterious***	***Deleterious***	***Deleterious***	***Deleterious***	***Deleterious***
PhD-SNP	***Disease***	***Disease***	Neutral	Neutral	Neutral	Neutral	***Disease***	***Disease***	***Disease***	***Disease***	***Disease***	***Disease***
SNPs&GO	***Disease***	***Disease***	Neutral	Neutral	Neutral	Neutral	***Disease***	***Disease***	***Disease***	***Disease***	***Disease***	***Disease***
MutPred^3^	***0*.*879***	***0*.*979***	0.275	*0*.*467*	0.32	0.302	***0*.*681***	***0*.*72***	***0*.*787***	***0*.*756***	***0*.*678***	***0*.*688***
MutationAssessor	NA	***High***	NA	Neutral	NA	Neutral	NA	*Medium*	NA	***High***	NA	*Medium*
FatHMM-Unweighted	NA	***Damaging***	NA	Tolerated	NA	Tolerated	NA	***Damaging***	NA	Tolerated	NA	Tolerated
FatHMM-Weighted	NA	***Damaging***	NA	***Damaging***	NA	***Damaging***	NA	***Damaging***	NA	***Damaging***	NA	***Damaging***
MutationTaster-2	NA	***Disease Causing***	NA	Polymorphism	NA	Polymorphism	NA	***Disease Causing***	NA	***Disease Causing***	NA	***Disease Causing***
Likelihood Ratio Test	NA	***Delererious***	NA	Neutral	NA	Neutral	NA	***Delererious***	NA	***Delererious***	NA	***Delererious***
Condel	NA	***Delererious***	NA	***Delererious***	NA	***Delererious***	NA	***Delererious***	NA	***Delererious***	NA	***Delererious***

Descriptions of prediction are conforming each analysis tool. The predicted impact of amino acid changes on b^o,+^AT protein is depicted with different font: both bold and italic, severe; only italic, medium; neither bold and italic, light or none. Amino acids are depicted with symbols recommended by IUPAC-IUB.

^1^reported homologous variants in humans

^2^score ranged from C0 (Low risk) to C65 (High risk)

^3^score showing probability of deleterious variant;

NA, not available.

Of the intronic variants in the *SLC7A9* gene, a heterozygous single nucleotide variant was identified at a highly conserved nucleotide (observed in multiple vertebrate nucleotide alignments) at the splice donor site of intron 10 (Fig 1) in a DSH cat (#4). Furthermore, a homozygous intronic variant, identified in another cystinuric cat (#5), was located at one of 2 putative branchpoints in intron 11 that is predicted to be critical based on the branch site motif sequence (Fig 1). We were unable to detect enough transcription of the *SLC7A9* gene in blood samples to be able to successfully sequence the mRNA from either control or cystinuric cats, but expression of the *SLC3A1* and a house-keeping gene could be demonstrated in blood from these and other cats (data not shown).

In addition to the *SLC7A9* variants, 6 exonic and 10 noncoding variants were observed in the *SLC3A1* gene of 6 cystinuric cats relative to the published feline sequence (GenBank accession #: AANG02047288.1, AANG02047289.1 and AANG03012118.1) and the sequenced normal cat (S2 Table). None of the cats had the disease-causing missense *SLC3A1* variant previously reported [6]. Aside from the reported missense variants in *SLC3A1* [6], another *SLC3A1* missense variant (c.1798C>G; p.Pro600Ala) was identified. However, this variant was located in a non-conserved rBAT protein region predicted to be well tolerated by most models. Thus, no new *SLC3A1* variants causing cystinuria in cats were discovered among the cats studied here.

Genotyping tests were developed to screen for the 3 deleterious missense variants, the putative splice site and the branchpoint in 150 non-cystinuric non-purpose-bred and purebred control cats from the United States using the polymerase chain reaction (PCR)-restriction fragment length polymorphism (RFLP) and real-time PCR assays; none of the cats were found to have any of the identified mutant *SLC7A9* alleles. However, an additional cystinuric DMH (#1C) cat was found to be homozygous for the c.881T>A (p.Val294Glu) variant seen in the 2 purebred cats of this report.

## Discussion

Cystinuria, recognized by Sir Archibald Garrod more than a century ago, was one of the first identified inborn errors of metabolism in humans. Because cystine calculi obstructing the urinary tract are readily recognized, cystinuria has also been discovered in pet dogs [2–4] and cats [5–7] as well as ferrets [9] and several wild animals [8,11–13]. In fact, cystinuria is common in many canine breeds and some wild carnivore species and also occurs frequently in humans (1: 7,000) [1]. A spontaneous cystinuria murine model has also recently been described (129S2/SvPasCrl) (p.Glu383Lys in *SLC3A1*) and 3 knockout murine models (C3HeB/FeJ-MRL/MpJ, 129P2/OlaHsd-57BL/6J) (p.Asp140Gly in *SLC3A1*, disruption of exon 3 to 9 in in *SLC7A9*, compound heterozygotes of the two variants [C3HeB/FeJ/MRL/MpJ-129P2/OlaHsd/57BL/6J]) have been generated [10,22–24].

The subunits of the major cystine and dibasic amino acid transporter b^o,+^ in the proximal renal tubules are coded for by the two solute channel genes *SLC3A1* and *SLC7A9*. The rBAT and b^o,+^AT proteins are nearly the same size in all mammals (number of amino acids of rBAT/b^o,+^AT: 681/490 in cats, 685/487 in humans, 700/490 in dogs, 685/487 in mice) and the amino acid sequences of b^o,+^AT are highly conserved (humans 90%, dogs 95% and mice 89% homologous to cats) thereby allowing functional comparisons. While the molecular genetic basis for this disease has been described in many human patients [1], 4 murine models [10,22–24], 5 canine breeds (Raj et al. unpublished, 2016) [3], and one cat with an *SLC3A1* variant [6], we report here on the first 5 *SLC7A9* variants in cystinuric cats.

Human cystinuria is genetically heterogeneous with 169 *SLC3A1* (Type A) and 121 *SLC7A9* (Type B) variants found thus far in human patients (HGMD database accessed on April 2016) [25], respectively. Such molecular heterogeneity explains the varied disease features and also accounts for the autosomal recessive (type I) and dominant (type II) mode of inheritance. A similar heterogeneity has recently been reported in cystinuric dogs [3]. After reporting on the first variant in the *SLC3A1* gene causing cystinuria in a DSH cat [6], we subsequently examined 7 additional cystinuric cats of different breeds and from different geographical areas. Although no additional *SLC3A1* variants were found, we identified 3 disease-causing missense variants and one splicing and one putative banchpoint variant in the *SLC7A9* gene. As all of these affected cats were either female and/or neutered, an androgen-dependent type of cystinuria as described in dogs could be excluded. These feline studies confirm the molecular genetic heterogeneity of cystinuria. In fact, among the approximate 2 dozen hereditary diseases characterized at the molecular genetic level in cats, cystinuria is the most genetically diverse, similar to feline acute intermittent porphyria with 6 different variants in the *HMBS* gene [26,27].

The c.881T>A *SLC7A9* point variant substitutes a highly conserved, hydrophobic valine to a negatively charged, hydrophilic glutamic acid (p.Val294Glu). This *SLC7A9* missense variant is located in the extracellular domain of b^o,+^AT protein. A homologous variant has not yet been reported in other species, but will likely not be tolerated according to most models. Furthermore, despite the absence of an identified functional domain, variants in this region of *SLC7A9* (p.Leu283Phe and p.Ser286Phe) also cause cystinuria in human patients [28,29]. One Maine coon, one Sphynx and one DMH cat were found to be homozygous for this point variant, and they all developed urolithaisis when mature (after one year of age). These data suggest that the 3 breeds have a common ancestry. The longhaired Maine Coon breed was established in the United States in the 1860s, while the hairless Sphynx was developed a century later (in the 1960s) in Canada with crossing into Devon Rex cats [30]. While both Maine Coon and Sphinx are phylogenetically grouped together as Western European cats, no direct genetic relatedness exists between these 2 breeds [31,32]. The presence of the same variant in these different breeds in different geographical locations in North America suggests a more wide and common distribution of the mutant allele, but no larger surveys have been undertaken.

The c.706G>A *SLC7A9* point variant changes a highly conserved, negatively charged aspartic acid to an uncharged asparagine (p.Asp236Asn) in the cytoplasmic domain of b^o,+^AT protein. One DMH cat homozygous for this variant showed stranguria due to crystalluria as a juvenile and cystine uroliths formed recurrently during the next 4 years. While no other cystinuric cats with the same mutant allele have been found, a homologous missense variant (p.Asp233Glu) was reported in a 11 year old cystinuric child in Greece [33]. While the substitution of the aspartic acid at this position is predicted by models to be highly deleterious, this boy was a compound heterozygote with a splice site variant in the other *SLC7A9* allele. Although the relative contribution of each variant remains to be determined, variant of both *SLC7A9* alleles in the cystinuric cat and child suggests a recessive mode of inheritance.

The c.1175C>T *SLC7A9* point variant replaces a highly conserved, uncharged, hydrophilic threonine with a hydrophobic methionine (p.Thr392Met) in the tenth transmembrane domain of b^o,+^AT protein. A 4-month old DLH cat was homozygous for this missense variant. Furthermore, a healthy littermate was homozygous for the wild-type allele and did not develop cystine calculi over the 2 year observation period, supportive of the deleterious effect of this missense variant. The homologous variant (p.Thr389Met) was recently identified in a human cystinuric patient in the United Kingdom, albeit no further clinical information was available [34].

Interestingly the cystinuric kitten with p.Thr392Met *SLC7A9* missense variant in this report also developed intermittent neurological signs and died one month after diagnosis of cystinuria. While the cause of neurological impairment was not determined, one might speculate it was secondary to an arginine deficiency caused by impaired intestinal arginine absorption and excessive renal arginine excretion caused by the mutant *SLC7A9*. Arginine is an essential amino acid for cats and its deficiency can lead to hyperammonemia and neurological signs [35]. Similar neurologic signs were reported in another cystinuric cat with an *SLC3A1* variant [6].

While *SLC3A1* variants were observed in a previously reported cat (Type IA; p.Arg448Trp) and cause a recessively inherited cystinuria in 4 different canine breeds (Labrador Retrievers, Scottish Terriers, Newfoundlands and Landseers) (Type IA; p.Arg196*, p.Gly117Alafs*41) and a semi-dominant type (Type IIA; p.Thr366_Thr367del) cystinuria in Australian Cattle dogs, only one canine breed with an *SLC7A9* variant has been reported to date. All cystinuric Miniature pinschers in a large family were heterozygous for the p.Gly322Arg variant in the *SLC7A9* gene, indicating a dominant mode of inheritance, with potentially lethal homozygosity [3].

For many *SLC7A9* variants in humans, an autosomal dominant pattern can be observed with respect to COLAuria, but the penetrance of cystine calculi formation is incomplete [36]. In this report, no cystinuric cats were found to be heterozygous for any of the 3 missense variants, although this may be due to a lack of cystine crystal and calculi formation in asymptomatic heterozygous cats. Established simple variant screening tests will allow for expanded screening of other cats with cystine crystalluria and calculi-induced urinary tract obstruction and their asymptomatic relatives to determine if cats with heterozygous missense variants have cystinuria and are at risk for developing calculi.

Although the cats studied here had cystine crystals or calculi and cystin- and COLAuria, further efforts to functionally assess specific missense variants await future studies. Unfortunately, in silico structural analysis of the b^o,+^AT protein mutants could not be undertaken, due to incomplete knowledge of the normal b^o,+^AT structure. However, we did assess the effects of these variants on the b^o,+^AT and rBAT proteins (including the previously reported variant in a cat) using multiple available prediction programs (Table 2) to reduce the chance of false predictions [37]. In addition, we analyzed the effects of equivalent amino acid substitutions in the human proteins as well as amino acid changes predicted to be inconsequential in feline rBAT. Although the programs basically assess sequence conservation using multiple sequence alignments and some programs including PolyPhen2, SNPs&GO, MutPred and PhD-SNP also combine homology information with various types of structural and functional annotations of the proteins, such as amino acid properties, the location of functional sites, and the secondary structure and membrane topology of the protein, each program uses its specific algorithm which sometimes causes different predictions [37–40]. When used together in this study, the programs were in good agreement regarding the deleterious effects of the missense variants observed in cystinuric cats and their homologues in humans.

Two additional cystinuric cats had no exonic *SLC7A9* variants, but one mildly cystinuric cat was heterozygous for a unique variant at the splice donor site in intron 10, and the other cystinuric cat was homozygous for a unique variant at a putative branchpoint nucleotide in intron 11. Those nucleotides and motifs are typically highly conserved among species [41]. Thus, these variants are expected to affect the normal formation of mRNA. However, kidney tissues were not available from either cystinuric cats and attempts to examine *SLC7A9* mRNA in blood unfortunately failed, even for the control cat. In addition, it remains unclear whether the heterozygous variant acts in a semi-dominant manner or whether there was a second unidentified variant that affected the function of the second allele. Thus, delineating the pathological relevance of these variants must await future studies.

In conclusion, we describe here novel models of cystinuria in a diversity of feline breeds, with unique *SLC7A9* variants and clinical features. Based on our findings, cystinuric cats can now be screened for several *SLC7A9* variants, providing beneficial insight for both veterinarians and cat breeders. In addition, such cats may serve as valuable large animal disease models to further characterize cystinuria and amino acid deficiency in disease and also to test novel therapies like the administration of L-cystine dimethyl ester to prevent large calculi formation and urinary tract obstruction [42,43].

## Materials and Methods

### Animals and samples

Cats with cystine crystals and/or uroliths from different regions in North America were included in these studies (Table 1). Voided urine and ethylenediaminetetraacetic acid (EDTA)-anticoagulated blood samples were sent chilled or frozen to the Metabolic Genetics Laboratory at the University of Pennsylvania after acquiring written informed owner consent. Archival urine from research colony cats at the University of Pennsylvania and archived DNA samples from cats from the United States were used as controls. These studies were approved by the Institutional Animal Care and Use Committee of the University of Pennsylvania (A3079-01).

### Biochemical methods of urine analysis

Urinary cystine and COLA concentrations were determined by qualitative cyanide-nitroprusside test [44] and quantitative amino acid analyzer (Biochrom 30+ Aminoacid Analyser, Biochrom Ltd., Cambridge, UK) [45]. Quantitative crystallographic analysis of calculi was performed by the Hill’s Minnesota Urolith Center, University of Minnesota [46].

### Genetic analyses

Genomic DNA was extracted from EDTA blood of cystinuric cats and from buccal swabs or EDTA blood of non-cystinuric cats using standard DNA extraction kits. Genomic DNA covering all exons and intronic flanking regions (*SLC3A1*: minimum ≥33 bp, average 125 bp; *SLC7A9*: minimum ≥73 bp, average 121 bp) of the feline *SLC3A1* and *SLC7A9* genes were amplified by PCR with exon flanking primers using reagents and procedures as previously described [6]. While primers for the *SLC3A1* gene amplification were as previously described [6], primers for the *SLC7A9* gene were newly designed based upon the Felis catus-6.2 feline reference genome assembly (S1 Table).

Following amplification, each PCR product together with a loading dye was electrophoresed on a 2% agarose gel containing ethidium bromide. Under UV light exposure, observed DNA bands were excised and purified using a QIAquick Gel Extraction kit (Qiagen, Valencia, CA), and analyzed by Sanger sequencing with PCR primers at the University of Pennsylvania’s Core DNA Sequencing Facility on an Applied Biosystems instrument. The sequences were compared to the feline reference and control genome sequence using the NCBI-Blast-tool. All exonic and intronic flanking as well as the 5’ and 3’ untranslated regions of both genes were analyzed.

The deduced amino acid sequences for rBAT and b^o,+^AT of the cystinuric cats were aligned with those of one healthy cat, the reference feline genome sequence, and other vertebrates using ClustalW [47], and further assessed by multiple programs to determine the effect of any amino acid variants (Table 2). Genetic nomenclature used is in accordance with the Guidelines and Recommendations for Mutation Nomenclature by the Human Genome Variation Society [48].

For *SLC7A9* genotyping, PCR-RFLP or real-time PCR assays for 3 deleterious missense and other 2 intronic variants were established to screen control group cats and an additional cystinuric domestic medium-hair (DMH) cat which were not sequenced. The PCR product of exon 7 in the *SC7A9* gene was digested for 2 hours at 37°C with the restriction enzyme *BceAI*, and then separated by electrophoresis on a 6% polyacrylamide gel. For the real-time PCR assays, allele specific TaqMan MGB probes and dedicated primer pairs were used to detect the variants in exon 5 and 10 and intron 10 and 11 of the *SLC7A9* gene (S1 Table).

## Supporting Information

S1 Table Primer and probe pairs, length of DNA product, and annealing temperatures used for the amplification of the *SLC7A9* exons and real-time PCR assays. PCR, polymerase chain reaction; RFLP, restriction fragment length polymorphism; bp, base pair.
(XLSX)

S2 Table Variants in exons and the flanking intronic regions of the *SLC3A1* and *SLC7A9* gene identified in healthy and cystinuric cats compared to reference sequence. There were 6 and 27 variants in the *SLC3A1* and *SLC7A9* gene between the healthy cat and feline reference sequence. In contrast, when compared with the feline reference sequence and the healthy cat sequence, cystinuric cats had 16 nucleotide changes in the *SLC3A1* gene sequence; 2 missense and 4 silent variants in coding regions, and 9 variants and 1 insertion in the flanking regions of several exons. The cystinuric cats also had 51 nucleotide changes in the *SLC7A9* gene sequence; 3 missense and 7 silent variants in coding regions, and 37 variants and 4 deletion in the flanking regions of several exons. Variants unique to cystinuric cats are depicted with bold letters. Amino acids are depicted with symbols recommended by IUPAC-IUB. DSH, domestic short-haired cat; DLH, domestic long-haired cat; DMH, domestic middle-haired cat; NA, nucleotides undetected due to sequencing problem, ^1^GenBank accession number (AANG02047288.1, AANG02047289.1 and AANG03012118.1 in *SLC3A1* gene; AANG03028500.1 and AANG03028501.1in *SLC7A9* gene), ^2^cystinuric cat previously reported [6].
(XLSX)
